# Supplementary material for: Type I interferon drives T cell cytotoxicity by upregulation of interferon regulatory factor 7 in autoimmune kidney diseases in mice
Source: Nat Commun. 2025 May 20;16:4686. doi: 10.1038/s41467-025-59819-7 (PMC12092595; doi:10.1038/s41467-025-59819-7)
Supplement: Supplementary file 1 — Supplementary Information [file 41467_2025_59819_MOESM1_ESM.pdf]

Supplementary Information

Type I interferon drives T cell cytotoxicity by upregulation of interferon regulatory factor 7 in autoimmune kidney diseases in mice

Huiying Wang[1,2], Jonas Engesser[1,2], Robin Khatri[2,3], Darius P. Schaub[2,3], Hans-Joachim Paust[1,2], Zeba Sultana[1,2,3], Saskia-Larissa Jauch-Speer[1], Anett Peters[1], Anna Kaffke[1], Stefan Bonn[2,3,4], Tobias B. Huber[1,2,4], Hans-Willi Mittrücker[2,5], Christian F. Krebs[1,2,4], Ulf Panzer[1,2,4*], Nariaki Asada[1,2*]

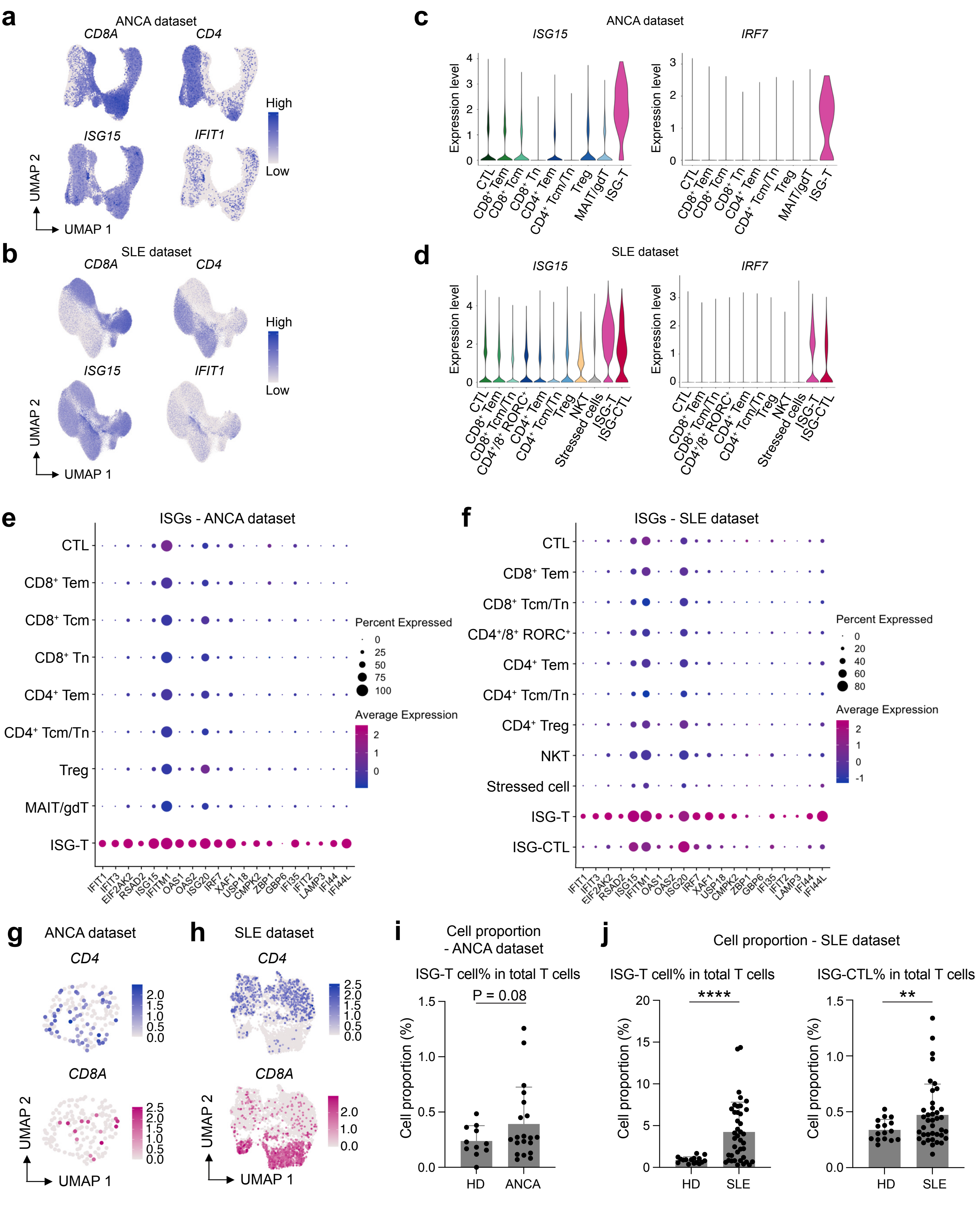

Supplementary Fig. 1: scRNA-seq analysis of blood T cells in AAV and SLE patients.

a, **b** UMAP plots showing the expression of *CD8A*, *CD4*, *ISG15*, and *IFIT1* in the scRNA-seq dataset of patients with AAV (a) and SLE (b).

c, **d** Violin plots showing the expression of *ISG15* and *IRF7* in AAV (c) and SLE (d) patients.

e, **f** Dot plots depicting the expression of ISGs in ISG-T cells from the ANCA dataset (e) and the SLE dataset (f).

g, **h** UMAP plots showing the expression of *CD4* and *CD8A* in reclustered ISG-T cells from the ANCA dataset (g) and the SLE dataset (h).

i, **j** Bar graphs showing the ISG-T cell percentage in total T cells in the ANCA dataset (i) and SLE dataset (j).

i, j, P values were calculated by the unpaired two-tailed t-test with Welch's correction.

(* $p < 0.05$, ** $p < 0.01$, *** $p < 0.001$, **** $p < 0.0001$)

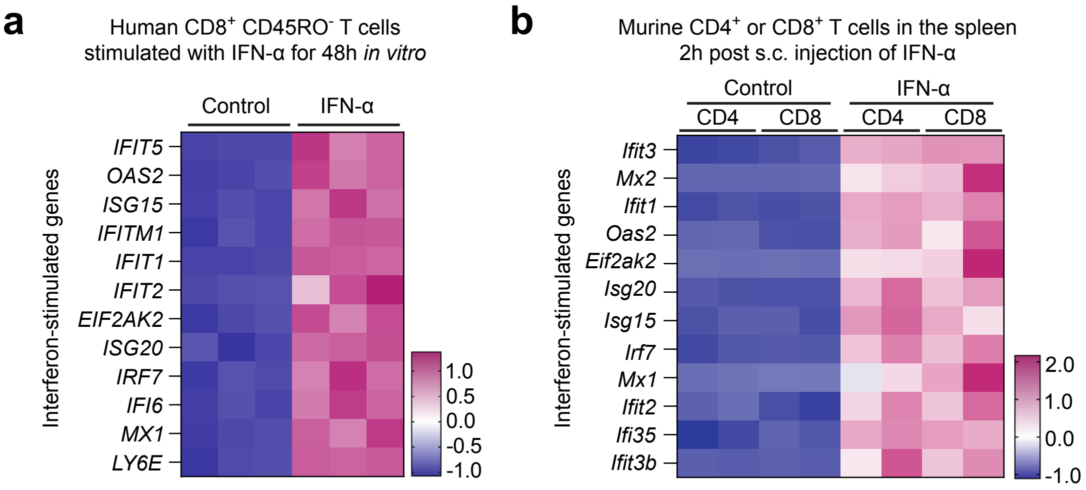

Supplementary Fig. 2: T cell stimulation with IFN-I leads to the expression of interferon-stimulated genes.
a Analysis of transcriptome data GSE17302. Human CD8^{+} CD45RO^{-} naive T cells from three healthy donors were stimulated with IFN-α for 48 hours and analyzed by microarray. A heatmap shows the expression of interferon-stimulated genes.
b Analysis of transcriptome data GSE75202. Mice were subcutaneously injected with IFNα two hours prior to the analysis of spleen CD4^{+} or CD8^{+} T cells using microarray. A heatmap shows the expression of interferon-stimulated genes.

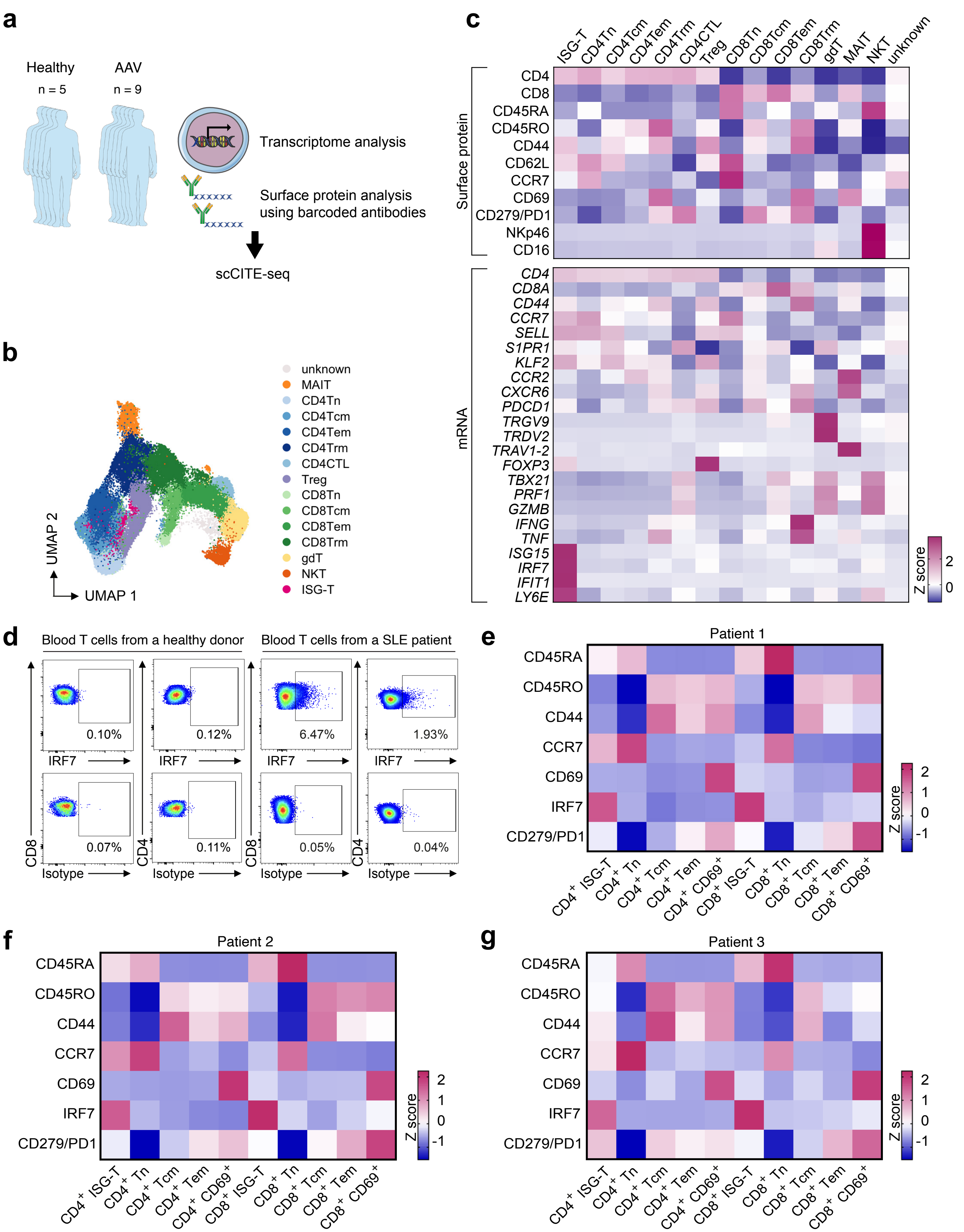

Supplementary Fig. 3: Surface marker analysis of ISG-T cells from patients with AAV or SLE.
a T cells from AAV patients and healthy donors were analyzed with scCITE-seq.
b UMAP plot showing different clusters.
c Heatmap showing the surface protein levels as well as marker genes expression in each cluster.
d Representative flow cytometry plots of blood T cells from a healthy donor and a patient with SLE.
e-g Heatmaps showing the expression of surface markers across different T cell subsets in each SLE patient. Z score was clculated based on mean fluorescence intensity.

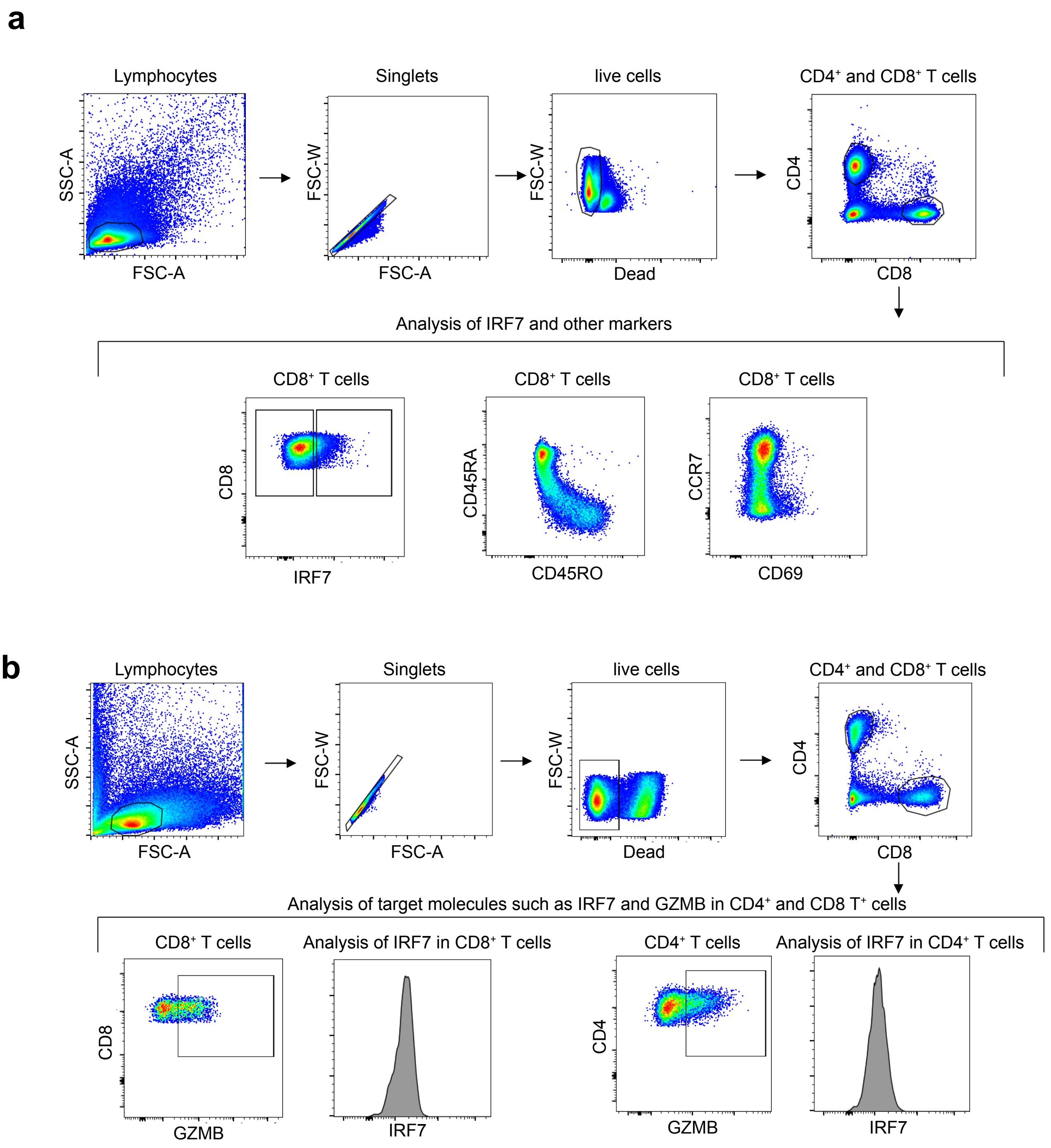

Supplementary Fig. 4: Examples of gating strategies and flow cytometry plots.

a Example of flow cytometry plots and gating strategy for analyzing blood T cells from patients with SLE. For both CD4$^+$ and CD8$^+$ T cells, gating and analysis were performed similarly.

b Example of flow cytometry plots and gating strategy for analyzing murine T cells.

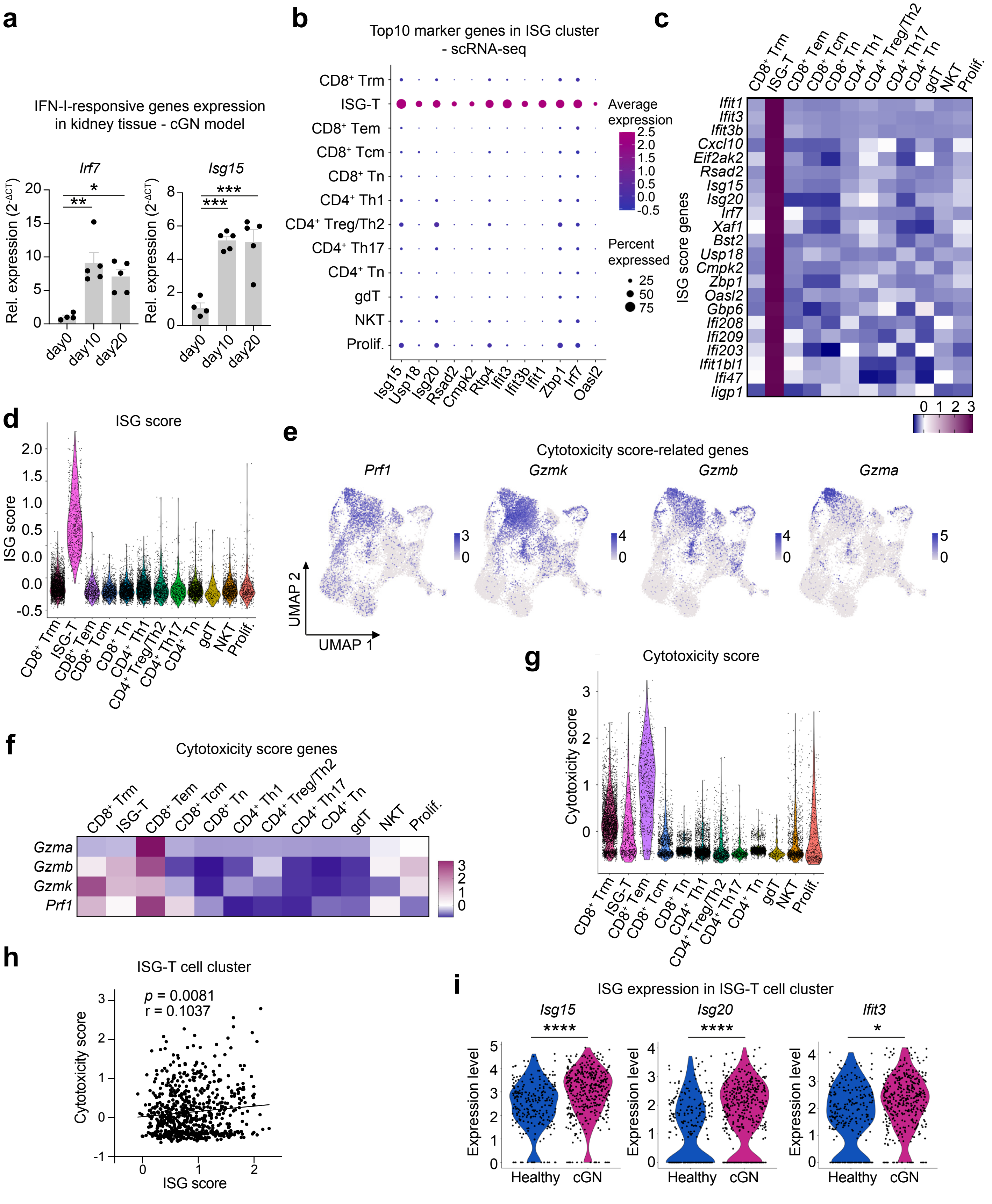

Supplementary Fig. 5: ISG and ISG-T cells in murine cGN model.
a RT-PCR analysis of renal *Irf7* and *Isg15* expression at days 0, 10, and 20 following cGN induction.
b Dot plot showing the expression of top 10 highly expressed marker genes in ISG-T cell cluster.
c The expression of ISG score genes in each cluster. **d** Violin plot showing the expression of ISG score.
e UMAPs showing the expression of cytotoxicity score genes expression.
f Heatmap showing the expression of cytotoxicity score genes.
g Violin plot showing the levels of cytotoxicity score.
h Scatter plot showing ISG score and Cytotoxicity score.
i Violin plots showing the expression of *Isg15*, *Isg20,* and *Ifit3*.
a, P values were calculated by one-way ANOVA with Tukey's multiple comparison test.
h, P value was calculated using Pearson correlation.
i, P values were calculated by unpaired two-tailed t-test with Welch's correction.
(* $p < 0.05$, ** $p < 0.01$, *** $p < 0.001$, **** $p < 0.0001$)

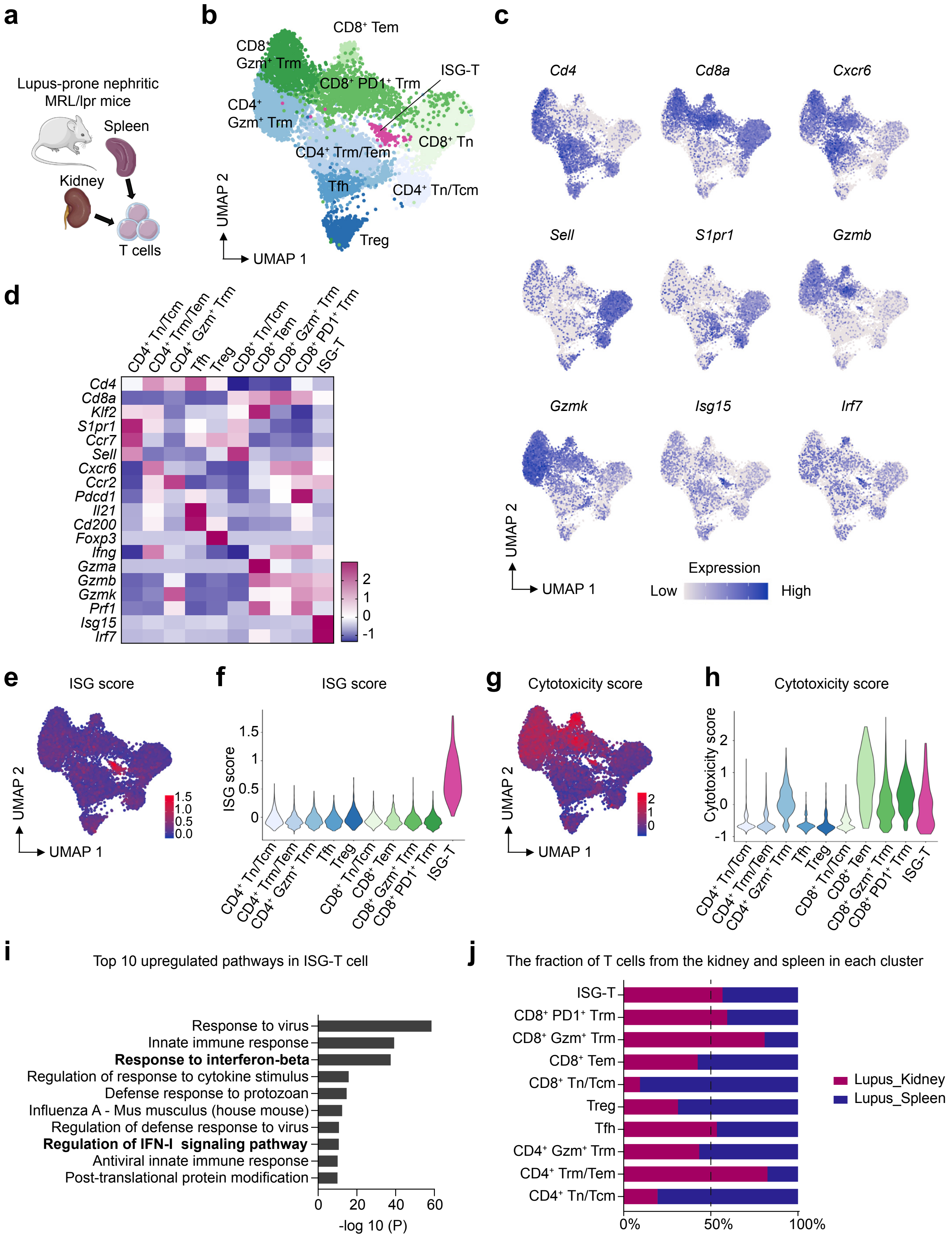

Supplementary Fig. 6: scRNA-seq of T cells in MRL/lpr lupus nephritis mouse.

a scRNA-seq dataset of T cells isolated from the nephritic kidney and the spleen was analyzed.

b UMAP plot showing the different T cell clusters.

c UMAP plots showing the expression of marker genes.

d Heatmap showing the expression of marker genes in different clusters.

e-h UMAP and violin plots showing the ISG score (**e** and **f**) and cytotoxicity score (**g** and **h**), respectively.

i Bar graph showing the top 10 upregulated pathways in ISG-T cells.

j Bar graph showing the fraction of T cells from the kidney and spleen in each cluster.

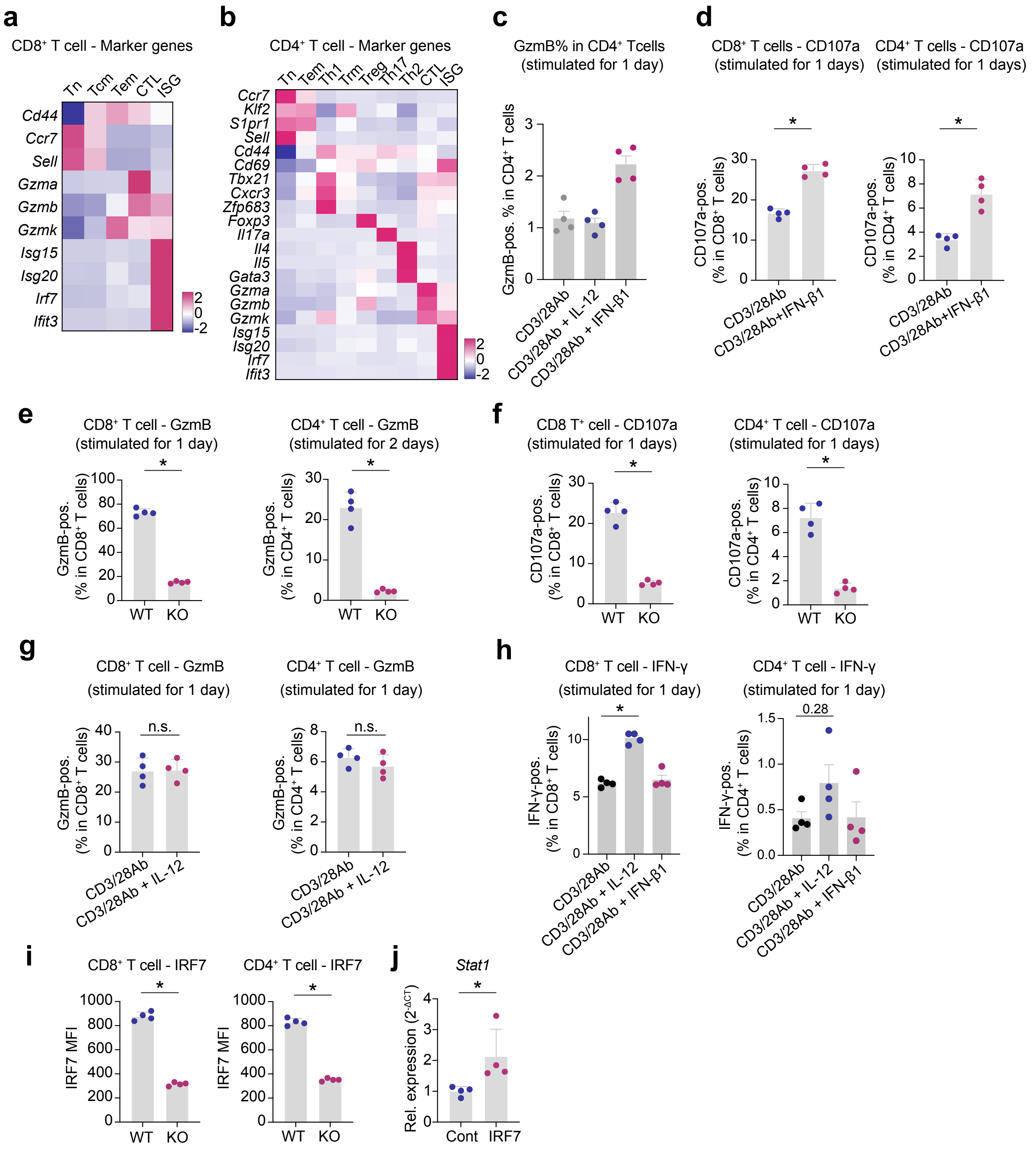

Supplementary Fig. 7: IFN-I induces cytotoxicity in T cells.

a, **b** Heatmaps showing the marker gene expression in CD8⁺ (**a**) and CD4⁺ (**b**) T cells.

c Bar graph showing the frequency of GzmB-producing CD4⁺ T cells.

d Bar graphs showing the frequencies of surface CD107a-positive CD8⁺ and CD4⁺ T cells.

e GzmB production in CD8⁺ and CD4⁺ T cells from wildtype and *Ifnar*1 KO mice.

f Surface CD107a expression in CD8⁺ and CD4⁺ T cells from wildtype and *Ifnar*1 KO mice.

g GzmB production in T cells stimulated with CD3/28Ab in the presence of vehicle or IL-12.

h IFN-γ production in T cells stimulated with CD3/28Ab in the presence of vehicle, IL-12, or IFN-β1.

i IRF7 levels in WT and *Ifnar1* KO T cells stimulated with CD3/28Ab in the presence IFN-β1 for 1 day.

j Bar graph showing *Stat1* expression levels in T cells one day post-transduction.

Cont, dominant-negative IRF7; IRF7, constitutively active IRF7.

d-g,i, j P values were calculated by Mann-Whitney test.

h, P values were calculated by Kruskal-Wallis test.

Data are mean + S.E.M. (* $p < 0.05$, ** $p < 0.01$)

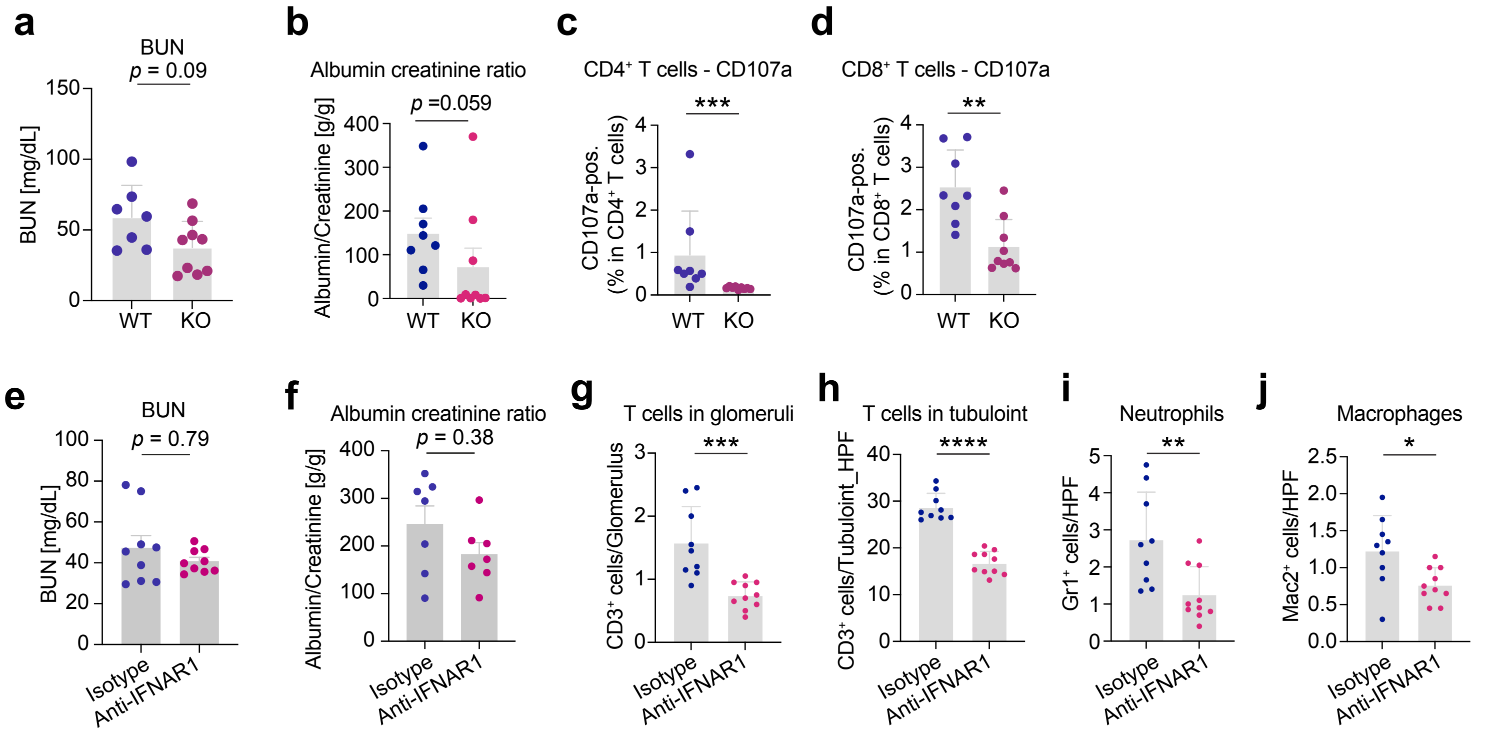

Supplementary Fig. 8: Targeting IFN-I signaling *in vivo*.

a-d Bar graphs showing the levels of BUN (**a**), albuminuria (**b**), and surface CD107a expression on $CD4^+$ T cells (**c**) and $CD8^+$ T cells (**d**) in *Ifnar1* KO and wildtype T cells.

e, **f** Quantification of albuminuria (**e**) and BUN (**f**) in anti-IFNAR1 Ab or isotype Ab-treated mice.

g-j Immune cells in the kidneys were quantified using immunohistochemistry.

P values were calculated by Mann-Whitney test. Data are mean + S.E.M (* $p < 0.05$, ** $p < 0.01$, *** $p < 0.001$).

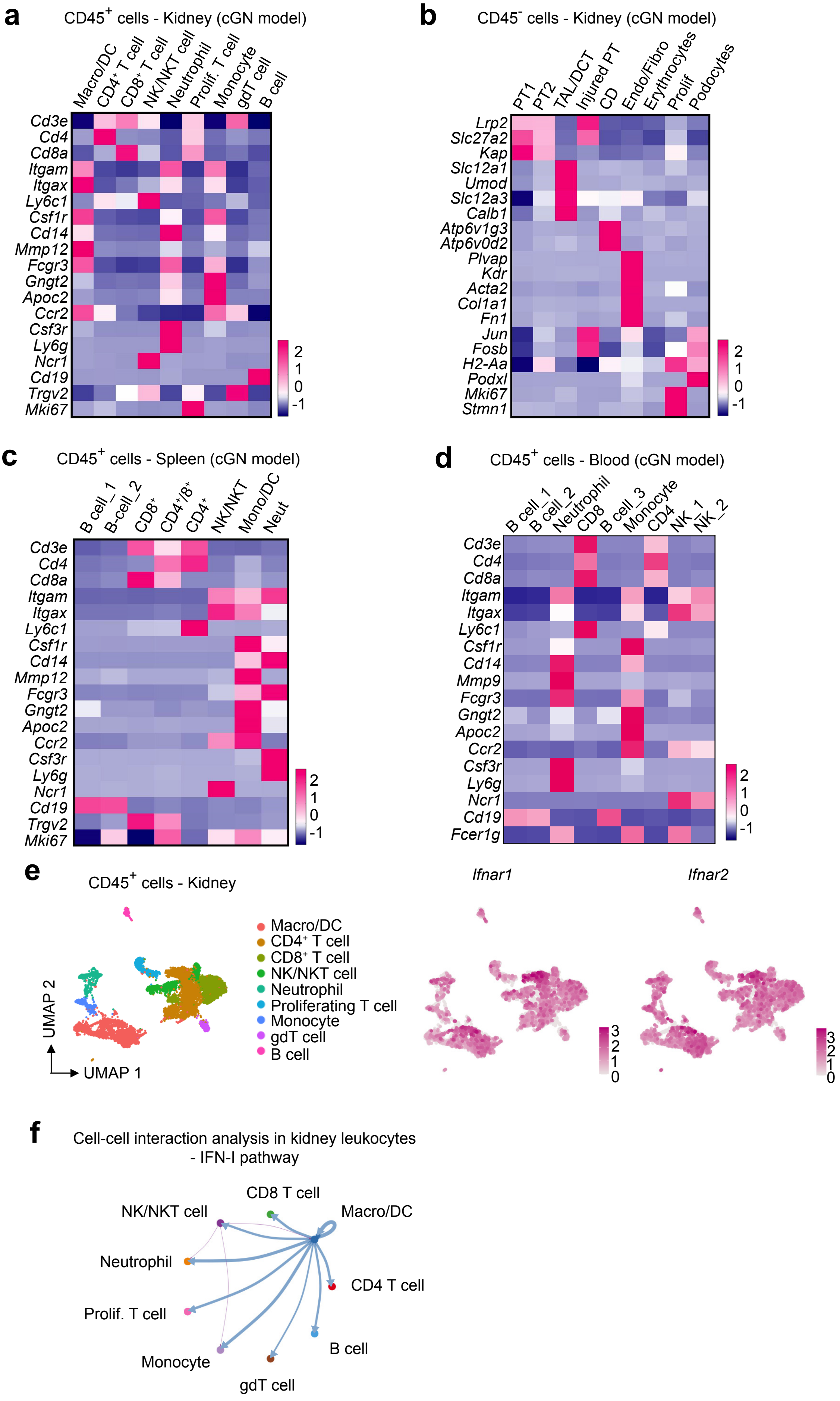

Supplementary Fig. 9: IFN-I is produced by kidney macrophage/dendritic cells.

a-d Heatmaps showing the marker gene expression of different cell types in kidney CD45$^+$ cells (a), kidney CD45$^-$ cells (b), spleen CD45$^+$ cells (c), and blood CD45$^+$ cells (d) datasets.

e UMAP plots showing the annotated clusters and the expression of *Ifnar1* and *Ifnar2* in the CD45$^+$ cells isolated from the nephritic kidneys.

f Cell-cell interaction analysis using CellChat, showing IFN-I signaling between kidney CD45$^+$ cells.

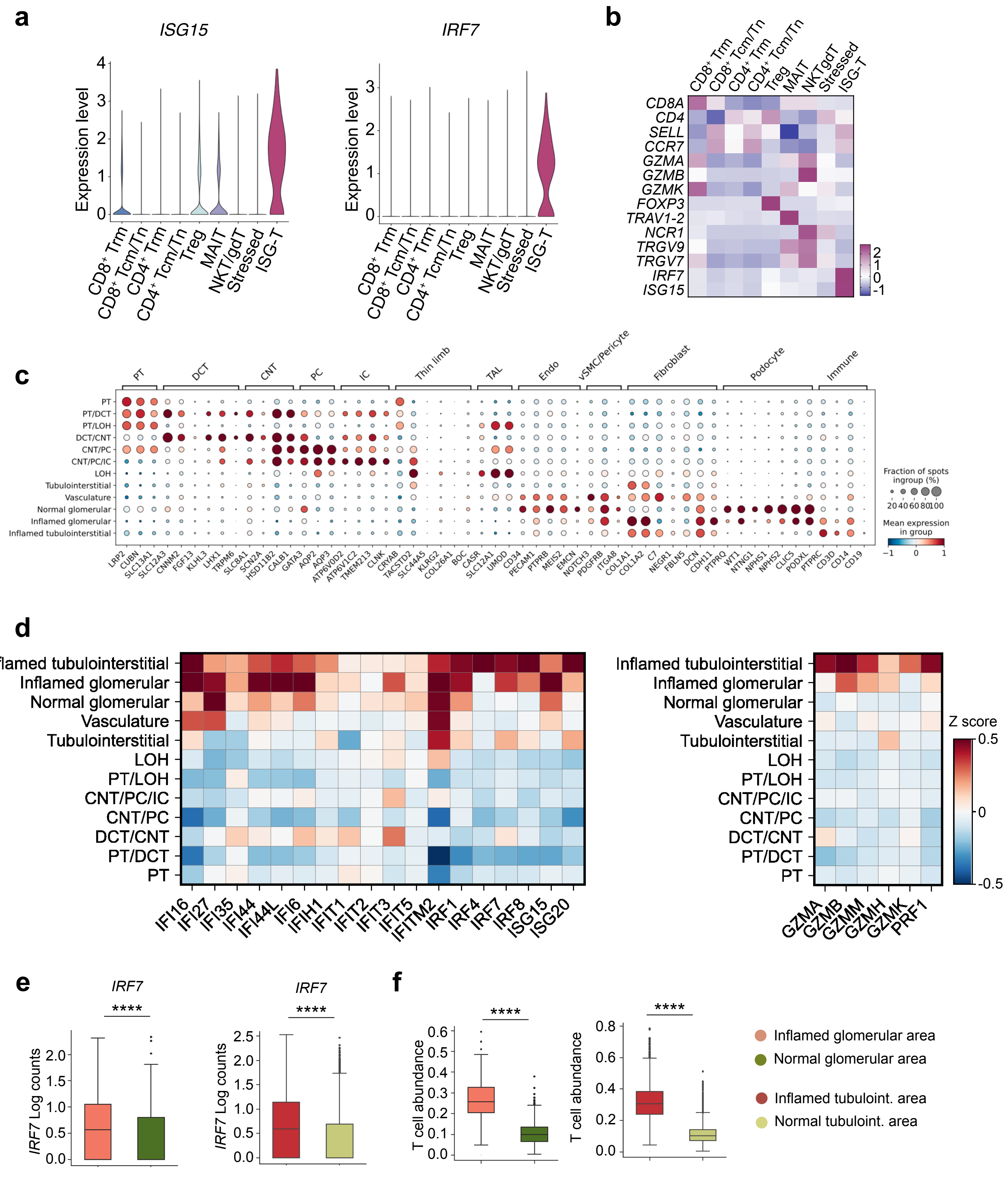

Supplementary Fig. 10: Transcriptome analysis of ANCA-GN.

a Violin plots showing the expression of *ISG15* and *IRF7* in kidney T cell clusters.

b Heatmap showing the expression of marker genes.

c Dot plot showing the expression of marker genes.

d Heatmaps showing the expression of ISGs and cytotoxic molecule genes.

e Graphs showing the expression of *IRF7*.

f Graphs showing the abundance of T cells calculated by deconvolution analysis.

a, b scRNA-seq, **c-f** spatial transcriptomics (*Visium*).

e, f P values were calculated by the unpaired two-tailed t-test with Welch's correction (**** $p < 0.0001$).

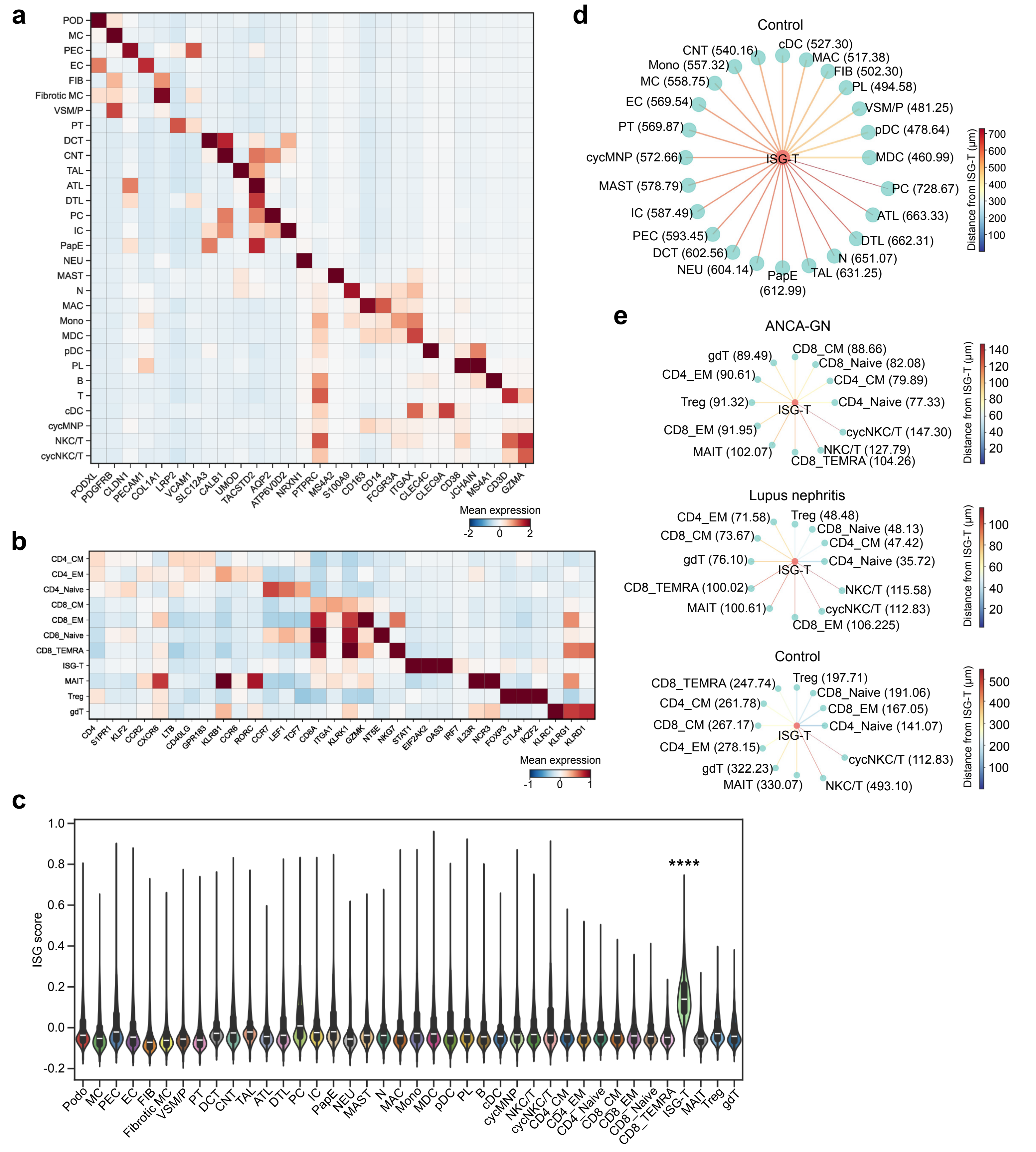

Supplementary Fig. 11: Imaging-based single-cell transcriptomic analysis of ANCA-GN and lupus nephritis.

a Heatmap showing the expression of marker genes of all cell types.

b Heatmap showing the expression of marker genes of T cell subtypes.

c Violin plot showing the levels of ISG score in different clusters. P value was calculated by the Wilcoxon rank-sum test. (**** $p < 0.0001$)

d Cell proximity analysis showing the distances from ISG-T cells to other non-lymphocyte cell types, with median distances shown in parentheses.

e Cell proximity analysis showing the distances from ISG-T cells to other lymphocytes, with median distances shown in parentheses.

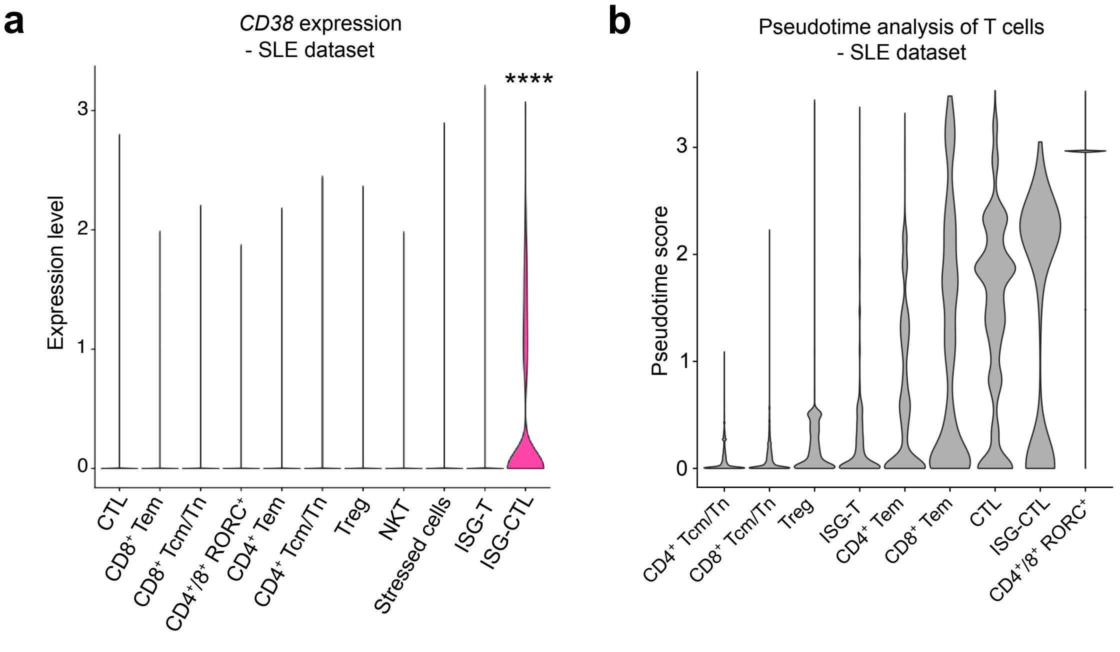

Supplementary Fig. 12: Characterization of ISG-CTL cluster in the SLE dataset.
a Violin plot showing the expression of *CD38* in the different T cell clusters.
P value was calculated by the Wilcoxon rank-sum test. (**** $p < 0.0001$)
b Pseudotime scores were calculated using Monocle 3, with $CD4^+$ and $CD8^+$ Tcm/Tn clusters set as the root of differentiation. While ISG-T cell cluster exhibited a relatively lower pseudotime score, ISG-CTL cluster showed a higher pseudotime score.

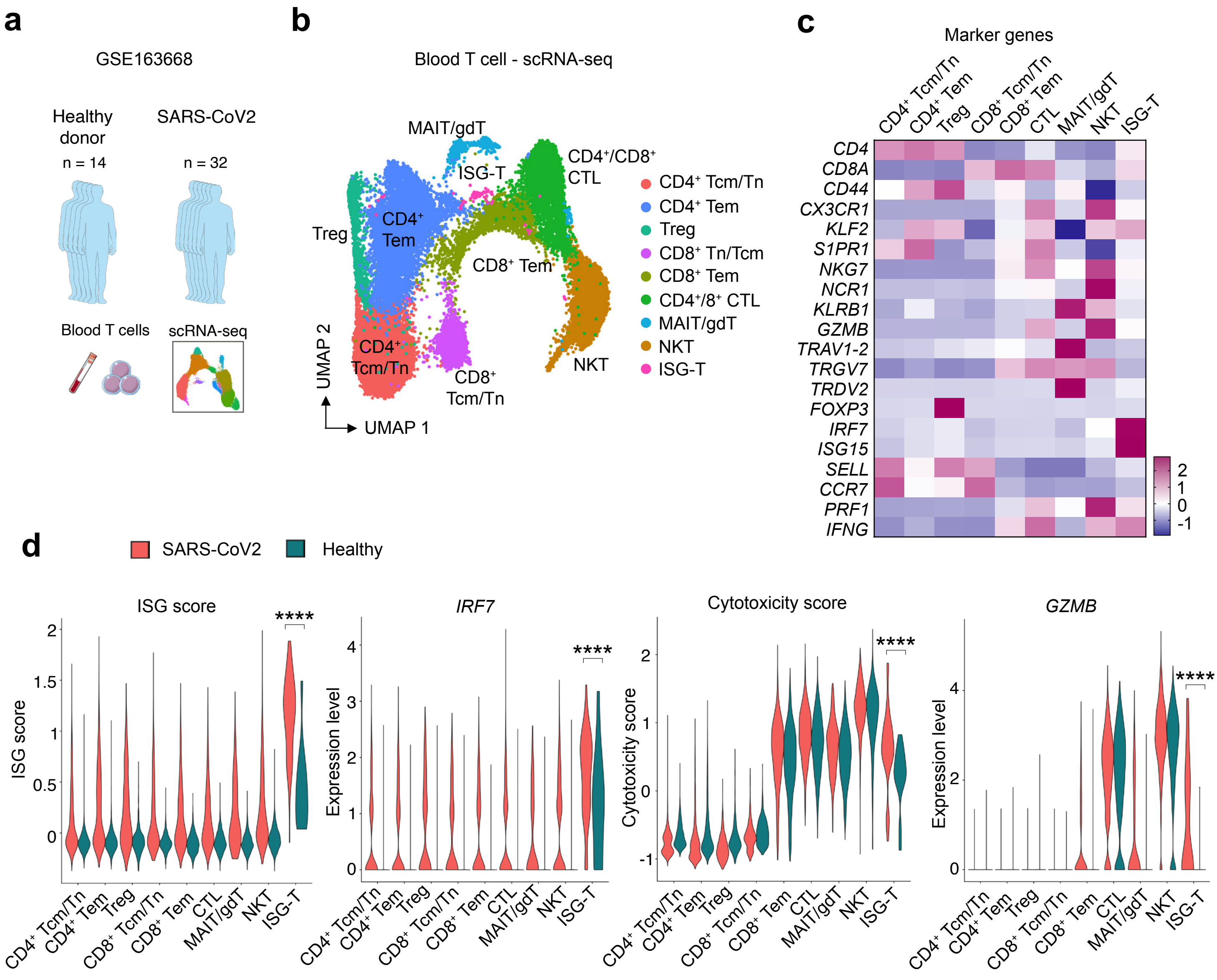

Supplementary Fig. 13: scRNA-seq analysis of T cells from patients with SARS-CoV2.

a scRNA-seq datasets of blood T cells from patients with SARS-CoV2 and healthy donor were analyzed.

b UMAP plot showing the different T cell clusters.

c Heatmap showing the marker gene expression in each cluster.

d Violin plots showing the levels of ISG score, *IRF7* mRNA expression, cytotoxicity score, and *GZMB* mRNA expression.

P values were calculated by one-way ANOVA with Tukey’s multiple comparison test.

Data are mean + S.E.M. (**** $p < 0.0001$)

Name	Age	Sex	SLEDAI	MMF	OS	MTX	Plaquenil	Nephrology_Class	Crea
HD1	7	F	ND	ND	ND	ND	ND	ND	ND
HD2	8	F	ND	ND	ND	ND	ND	ND	ND
HD3	8	F	ND	ND	ND	ND	ND	ND	ND
HD4	8	F	ND	ND	ND	ND	ND	ND	ND
HD5	12	F	ND	ND	ND	ND	ND	ND	ND
HD6	13	F	ND	ND	ND	ND	ND	ND	ND
HD7	14	F	ND	ND	ND	ND	ND	ND	ND
HD8	14	M	ND	ND	ND	ND	ND	ND	ND
HD9	16	F	ND	ND	ND	ND	ND	ND	ND
HD10	17	F	ND	ND	ND	ND	ND	ND	ND
HD11	18	F	ND	ND	ND	ND	ND	ND	ND
HD12	36	F	ND	ND	ND	ND	ND	ND	ND
HD13	39	F	ND	ND	ND	ND	ND	ND	ND
HD14	43	F	ND	ND	ND	ND	ND	ND	ND
HD15	46	F	ND	ND	ND	ND	ND	ND	ND
HD16	47	F	ND	ND	ND	ND	ND	ND	ND
HD17	50	F	ND	ND	ND	ND	ND	ND	ND
SLE1	10	F	0	0	0	0	1	ND	0.4
SLE2	12	F	6	0	1	0	0	ND	0.4
SLE3	12	F	4	0	0	0	0	Class II	0.4
SLE4	13	F	6	0	0	0	0	ND	0.5
SLE5	13	F	6	1	1	0	1	ND	0.7
SLE6	13	F	4	1	1	0	1	No biopsy as of 2018	0.4
SLE7	14	F	4	1	1	0	1	Class III (A)	0.4
SLE8	14	F	0	0	0	0	0	Class III	0.5
SLE9	15	F	19	0	0	0	0	ND	0.8
SLE10	16	F	6	1	1	0	1	Class II (A/C), Class V	0.6
SLE11	16	F	4	0	1	0	1	Class II	0.8
SLE12	16	F	2	0	1	0	1	ND	0.6
SLE13	16	F	4	1	1	0	1	Class V	0.5
SLE14	16	M	ND	1	1	0	1	Class V, Class III(C)	0.9
SLE15	16	M	4	1	1	0	1	Class IV-G (A)	1.5

SLE16	16	F	2	0	1	0	1	ND	ND
SLE17	16	F	0	0	0	0	0	Class V	0.7
SLE18	17	F	0	1	0	0	0	Class II, Class V	0.7
SLE19	17	F	8	0	0	0	0	Class II	0.8
SLE20	17	M	5	0	0	0	0	ND	0.9
SLE21	17	F	4	1	1	0	1	ND	0.6
SLE22	17	F	6	0	1	0	1	ND	0.6
SLE23	17	F	6	1	1	0	0	Class V	0.5
SLE24	17	F	8	0	1	0	1	ND	0.6
SLE25	17	F	ND	1	0	0	1	Class IV-G (A)	0.5
SLE26	17	F	0	1	0	1	1	ND	0.8
SLE27	17	F	0	1	1	0	1	Class II	0.6
SLE28	18	F	18	0	0	0	0	Class III A	0.6
SLE29	18	F	8	0	1	0	1	Class II	0.6
SLE30	18	F	2	1	1	0	1	Class II, Class V	0.5
SLE31	18	F	0	1	0	0	1	ND	0.8
SLE32	18	F	4	1	1	0	1	Class IV-S (A), Class V	0.8
SLE33	19	F	12	0	1	0	0	ND	1.2
SLE34	24	F	2	0	1	0	1	ND	0.7
SLE35	27	F	4	0	1	0	0	ND	0.6
SLE36	36	F	0	0	0	0	0	ND	1
SLE37	37	F	15	0	0	1	1	ND	0.9
SLE38	47	F	6	0	0	0	1	ND	0.9
SLE39	58	F	2	1	1	0	1	ND	0.8
SLE40	62	F	2	0	1	0	1	ND	0.9
SLE41	63	F	14	0	1	0	1	ND	0.7

Supplementary Table 1: Clinical characteristics of SLE patients. Clinical information of patients analyzed in the SLE dataset in Figure 1 is shown.

Names	Age	Gender	ANCA-GN	Medication	MPO-ANCA	PR3-ANCA	Treatment	BVAS	CREATININE
Control1	27	M	No	No	N.A.	N.A.	-	N.A.	ND
Control2	36	F	No	No	N.A.	N.A.	-	N.A.	ND
Control3	64	F	No	No	N.A.	N.A.	-	N.A.	ND
Control4	62	F	No	No	N.A.	N.A.	-	N.A.	ND
Control5	33	M	No	No	N.A.	N.A.	-	N.A.	ND
Control6	62	F	No	No	N.A.	N.A.	-	N.A.	ND
Control7	70	M	No	No	N.A.	N.A.	-	N.A.	ND
ANCA1	79	F	No	No	+	-	-	13	0.61
ANCA2	81	M	No	No	+	-	-	13	1.11
ANCA3	44	M	Yes	No	+	-	-	12	2.45
ANCA4	69	F	No	No	+	-	-	8	0.67
ANCA5	70	M	No	No	+	-	-	18	1
ANCA6	81	M	No	No	+	-	-	12	1.18
ANCA7	52	F	No	No	+	-	-	9	0.51
ANCA8	75	F	Yes	No	+	-	-	12	0.54

Supplementary Table 2: Clinical characteristics of ANCA patients. Clinical information of patients analyzed in the ANCA dataset in Figure 1 is shown.

	**Healthy**	**SLE**	**ANCA**
Sample N	21	32	21
Age (SD)	47.3 (11.5)	35.1 (13.3)	58.0 (13.8)
Sex (% female)	45	78	43
GFR (SD)	104 (31)	64 (39)	46 (31)

Supplementary Table 3: Clinical characteristics of ANCA-GN and lupus nephritis patients. Clinical information of patients analyzed in Figure 7 is shown.

Names	Age	Gender	ANCA type	Medication	CREATININE
P005	72	M	MPO ANCA	CYC+Steroids	3.4
P007	58	M	MPO ANCA	Steroid	6.3
P008	80	M	MPO ANCA	Steroid	4.3
P015	67	M	MPO ANCA	Steroid	6.8
P018	63	M	MPO ANCA	Steroid	4.7
P019	64	M	MPO ANCA	Steroid	2.1
P023	66	F	PR3 ANCA	Steroid	1.2
P025	82	M	MPO ANCA	Steroid	2.2
P028	58	F	PR3 ANCA	not treated	0.8
P029	64	M	PR3 ANCA	Steroid	1.9
P053	81	F	MPO ANCA	Steroid	0.95
P055	69	F	PR3 ANCA	Steroid	7.5
P059	79	F	MPO ANCA	Steroid	1.22
P060	59	F	MPO ANCA	RTX+steroid	1.7
P067	75	F	MPO ANCA	not treated	1.97
P070	74	F	PR3 ANCA	CYC+Steroids+PLEX	5.1
P088	57	M	MPO ANCA	not treated	1.55
P089	67	F	MPO ANCA	not treated	2.9
P100	70	M	MPO ANCA	CYC+Steroids	11.2
P103	56	F	MPO ANCA	Steroid	2.73
P108	65	M	PR3 ANCA	not treated	1.47
P115	55	M	MPO ANCA	CYC+Steroids	3.4
P118	81	M	PR3 ANCA	Steroid	4.76
P126	42	M	MPO ANCA	CYC+Steroids+PLEX	5.89
P129	67	M	MPO ANCA	CYC+Steroids+PLEX	2.08
P137	84	F	MPO ANCA	MPO ANCA	2.14
P139	61	F	PR3 ANCA	not treated	1.84

Supplementary Table 4: Clinical characteristics of ANCA-GN patients. Clinical information of patients analyzed by scRNA-seq in Figure 8 is shown.

						Induction				
	Age	sex	MPO	PR3	Crea	Steroid	RTX	CYC	RTX+CYC	Plex
P144	76	f		yes	2.08	x			x	
P139	61	f		yes	1.84	x		x		
P137	84	f	yes		2.14	x		x		
P129	67	m	yes		2.08	x		x		
P126	42	m	yes		5.89	x		x		x
P088	57	m	yes		1.55	x		x		
P089	67	f	yes		2.9	x		x		
P103	56	f	yes		2.73	x		x		
P108	65	m		yes	1.47	x		x		
P053	81	f	yes		0.95	x	x			
P055	69	f		yes	7.5	x		x		x
P029	64	m		yes	1.9	x	x			
P020	54	m		yes	1.7	x		x		x
P004	62	f		yes	4.5	x		x		
P050	34	m	yes		3.4	x	x			
P025	82	m	yes		2.2	x	x			
P118	81	m		yes	4.76	x			x	
P070	74	f		yes	5.1	x		x		x
P018	63	m	yes		4.7	x		x		
P023	66	f		yes	1.2	x	x			
P143	50	m	yes		1.92	x		x		
P100	70	m	yes		11.2	x		x		
P105	38	m	yes		1.38	x			x	
P059	79	f	yes		1.22	x		x		
P067	75	f	yes		1.97	x		x		
P028	58	f		yes	0.8	x	x			
P019	64	m	yes		2.1	x	x			
P068	61	m		yes	0.93	x		x		

Supplementary Table 5: Clinical characteristics of ANCA_GN patients. Clinical information of patients analyzed by sequencing-based spatial transcriptomics in Figure 8 is shown.

	ANCA-GN (n=32)	Lupus nephritis (n=19)
Age, median (IQR)	55.5 (51 – 64.75)	35 (29 – 42)
Sex		
Female, n(%)	11 (34.37)	14 (73.68)
Male, n (%)	21 (65.63)	5 (26.32)
Histology, n (%)	ANCA Renal Risk Score	Lupus-Nephritis Class
	low: 7 (24.14)	Class III: 7 (38.89)
	medium: 16 (55.17)	Class IV: 4 (22.22)
	high: 6 (20.69)	Class III+V: 5 (27.78)
		Class IV+V: 2 (11.11)
Laboratory values, median (IQR)		
Creatinine (mg/dl)	2.5 (1.78 – 3.84)	0.9 (0.66 – 1.4)
eGFR (ml/min)	25.79 (13.77 - 36.4)	89 (53 - 112)
ACR (mg/g)	1165 (378 - 2175)	1100 (210 – 2130)
Autoantibody levels (U/ml)	MPO: 88 (55.59 – 122.3)	dsDNA: 55 (13 - 379)
	PR3: 41 (1.47 – 145.3)	

Supplementary Table 6: Clinical characteristics of ANCA-GN and lupus nephritis patients. Clinical information of patients analyzed by imaging-based spatial transcriptomics in Figure 9 is shown.

Names	Age	Gender	ANCA type	Medication	CREATININE
C011	62	M	N.A.	N.A.	0.91
C012	62	F	N.A.	N.A.	1.1
C013	62	F	N.A.	N.A.	1.1
C015	57	M	N.A.	N.A.	0.85
C018	78	M	N.A.	N.A.	1.2
P053	81	F	MPO ANCA	Steroid	0.95
P055	69	F	PR3 ANCA	Steroid	7.5
P059	79	F	MPO ANCA	Steroid	1.22
P060	59	F	MPO ANCA	RTX+steroid	1.8
P067	75	F	MPO ANCA	not treated	1.97
P069	73	M	MPO ANCA	RTX	4.36
P070	74	F	PR3 ANCA	CYC+Steroids+PLEX	5.1
P088	57	M	MPO ANCA	not treated	1.55
P089	67	F	MPO ANCA	not treated	2.9

Supplementary Table 7: Clinical characteristics of AAV patients. Clinical information of patients analyzed by scCITE-seq in supplementary Figure 3 is shown.

SLE	N=3
Age, median (IQR)	33 (27-47)
Sex, n(%)	
Female	3 (100)
Male	0
Serostatus, n(%)	
ANA positive	3 (100)
dsDNA positive	1 (33.33)
Histology, n(%)	
Lupus nephritis	2 (66.67)
Laboratory values, median (IQR)	
Creatinine (mg/dl)	0.64 (0.49-0.69)
eGFR (ml/min)	120 (106-129)
ACR (mg/g)	30.6 (18-328)
CRP (mg/l)	73 (8-181)
C3 (mg/dl)	117 (109-152)
C4 (mg/dl)	26.2 (14.2-31.8)
Use of immunosuppression, n(%)	
Steroids	1 (33.33)
Hydroxychloroquine	3 (100)
Azathioprine	1 (33.33)
MTX	1 (33.33)
Belimumab	1 (33.33)

Supplementary Table 8: Clinical characteristics of SLE patients. Clinical information of patients analyzed in supplementary Figure 3 is shown.
