# Supplementary material for: Type I interferon drives T cell cytotoxicity by upregulation of interferon regulatory factor 7 in autoimmune kidney diseases in mice
Source: Nat Commun. 2025 May 20;16:4686. doi: 10.1038/s41467-025-59819-7 (PMC12092595; doi:10.1038/s41467-025-59819-7)
Supplement: Supplementary file 2 — Reporting Summary [file 41467_2025_59819_MOESM2_ESM.pdf]

nature portfolio

Corresponding author(s): Nariaki Asada
Ulf Panzer

Last updated by author(s): May 5, 2025

Reporting Summary

Nature Portfolio wishes to improve the reproducibility of the work that we publish. This form provides structure for consistency and transparency in reporting. For further information on Nature Portfolio policies, see our Editorial Policies and the Editorial Policy Checklist.

Statistics

For all statistical analyses, confirm that the following items are present in the figure legend, table legend, main text, or Methods section.

n/a	Confirmed	
☐	☒	The exact sample size (*n*) for each experimental group/condition, given as a discrete number and unit of measurement
☐	☒	A statement on whether measurements were taken from distinct samples or whether the same sample was measured repeatedly
☐	☒	The statistical test(s) used AND whether they are one- or two-sided<br>*Only common tests should be described solely by name; describe more complex techniques in the Methods section.*
☒	☐	A description of all covariates tested
☐	☒	A description of any assumptions or corrections, such as tests of normality and adjustment for multiple comparisons
☐	☒	A full description of the statistical parameters including central tendency (e.g. means) or other basic estimates (e.g. regression coefficient) AND variation (e.g. standard deviation) or associated estimates of uncertainty (e.g. confidence intervals)
☐	☒	For null hypothesis testing, the test statistic (e.g. *F*, *t*, *r*) with confidence intervals, effect sizes, degrees of freedom and *P* value noted<br>*Give P values as exact values whenever suitable.*
☒	☐	For Bayesian analysis, information on the choice of priors and Markov chain Monte Carlo settings
☒	☐	For hierarchical and complex designs, identification of the appropriate level for tests and full reporting of outcomes
☐	☒	Estimates of effect sizes (e.g. Cohen's *d*, Pearson's *r*), indicating how they were calculated

Our web collection on statistics for biologists contains articles on many of the points above.

Software and code

Policy information about availability of computer code

Data collection	FACS symphony<br>Zeiss LSM800 confocal microscope
Data analysis	FlowJo software 10.5.3<br>GraphPad Prism 8<br>R studio (R version 4.40), Jupyter Notebook (6.5.4)<br>ZEN software 3.2 (Carl Zeiss, Jena, Germany)<br>Cell Ranger software suite (version 5.0.1, from 10x Genomics)<br>Seurat (version 4.0.2)<br>10x Genomics Space Ranger (v2.0.1)<br>Scanpy (v1.9.3)<br>Harmony (v0.1.0)<br>scikit-learn (version 1.3.1)<br>Scipy (v1.11.3)<br>networkx (v3.2.1)<br>Baysor (version 0.6.2)

For manuscripts utilizing custom algorithms or software that are central to the research but not yet described in published literature, software must be made available to editors and reviewers. We strongly encourage code deposition in a community repository (e.g. GitHub). See the Nature Portfolio guidelines for submitting code & software for further information.

Data

Policy information about availability of data

All manuscripts must include a data availability statement. This statement should provide the following information, where applicable:

- Accession codes, unique identifiers, or web links for publicly available datasets
- A description of any restrictions on data availability
- For clinical datasets or third party data, please ensure that the statement adheres to our policy

Public datasets: scRNA-seq data of blood T cells from patients with AAV is available at Genomic Expression Archive (GEA) with accession code E-GEAD-635 [https://humandbs.biosciencedbc.jp/en/hum0416-v1]17. scRNA-seq data of blood T cells of SLE patients is available in the dbGaP database under phs002048.v1.p1 [https://www.ncbi.nlm.nih.gov/projects/gap/cgi-bin/study.cgi?study_id=phs002048.v1.p1]20. The human transcriptome data of CD8+ T cells stimulated with IFN-α is available in NCBI Gene Expression Omnibus (GEO) under GSE17302 [https://www.ncbi.nlm.nih.gov/geo/query/acc.cgi?acc=GSE17302]24. The transcriptome data of spleen CD4+ T cells and CD8+ T cells from mice treated with IFN-α is available in NCBI GEO under GSE75202 [https://www.ncbi.nlm.nih.gov/geo/query/acc.cgi?acc=GSE75202]25. Transcriptome data of kidney biopsies from ERCB are available in the NCBI GEO under GSE104954 [https://www.ncbi.nlm.nih.gov/geo/query/acc.cgi?acc=GSE104954]36. scRNA-seq datasets of blood T cells from patients with SARS-CoV2 and healthy donors are available in the NCBI GEO under GSE163668 [https://www.ncbi.nlm.nih.gov/geo/query/acc.cgi?acc=GSE163668]49.

Publicly available datasets previously reported from our group: Our scRNA-seq datasets of CD45+ leukocytes (CD3+ and CD3- cells) isolated from murine kidneys are available in the NCBI GEO under GSE200880 [https://www.ncbi.nlm.nih.gov/geo/query/acc.cgi?acc=GSE200880]19. Our scRNA-seq dataset of blood and kidney T cells from patients with ANCA-GN is available in NCBI GEO under GSE253633 [https://www.ncbi.nlm.nih.gov/geo/query/acc.cgi?&acc=GSE253633]71. Our sequencing- or imaging-based spatial transcriptomics datasets39,71 are available in NCBI GEO under GSE250138 [https://www.ncbi.nlm.nih.gov/geo/query/acc.cgi?acc=GSE250138] and GSE294965 [https://www.ncbi.nlm.nih.gov/geo/query/acc.cgi?acc=GSE294965].

Newly generated datasets for this study: The scRNA-seq datasets of CD3+ cells from healthy murine kidneys, CD45+ cells from the blood and spleen at day 10 of cGN, kidney cells from wild-type B6 mice at day 10 of cGN generated in this study are available in NCBI GEO under GSE296304 [https://www.ncbi.nlm.nih.gov/geo/query/acc.cgi?acc=GSE296304].

All data are included in the Supplementary Information or available from the authors. The raw numbers for charts and graphs are available in the Source Data file whenever possible. Source data for graphs are provided with this paper.

Research involving human participants, their data, or biological material

Policy information about studies with human participants or human data. See also policy information about sex, gender (identity/presentation), and sexual orientation and race, ethnicity and racism.

Reporting on sex and gender	No sex- and gender-based analyses have been performed because of low sample sizes.
Reporting on race, ethnicity, or other socially relevant groupings	Patient information is summarized in the supplementary patient information.
Population characteristics	Patient information is summarized in the supplementary patient information.
Recruitment	Human kidney tissues were obtained from individuals enrolled in the Hamburg GN Registry, the European Renal cDNA Bank, or the CRU 228 ANCA-GN cohort. Additionally, in some cases, matched blood samples from the corresponding patients were also examined.
Ethics oversight	The studies were approved by the Ethik-Kommission der Ärztekammer Hamburg (the local ethics committee of the Hamburg Chamber of Physicians), and conducted in compliance with the ethical principles outlined in the Declaration of Helsinki.

Note that full information on the approval of the study protocol must also be provided in the manuscript.

Field-specific reporting

Please select the one below that is the best fit for your research. If you are not sure, read the appropriate sections before making your selection.

☒ Life sciences ☐ Behavioural & social sciences ☐ Ecological, evolutionary & environmental sciences

For a reference copy of the document with all sections, see nature.com/documents/nr-reporting-summary-flat.pdf

Life sciences study design

All studies must disclose on these points even when the disclosure is negative.

Sample size	Sample sizes were determined based on prior research conducted in our laboratories to use sufficient numbers of mice or cells in each group.
Data exclusions	no data were excluded
Replication	all findings were confirmed twice or more

Randomization	Mice were matched for age and sex before randomization
Blinding	Investigators were aware of the group allocation because the treatment groups needed to be clear when performing the experiments.

Reporting for specific materials, systems and methods

We require information from authors about some types of materials, experimental systems and methods used in many studies. Here, indicate whether each material, system or method listed is relevant to your study. If you are not sure if a list item applies to your research, read the appropriate section before selecting a response.

Materials & experimental systems

n/a	Involved in the study
☐	☒ Antibodies
☐	☒ Eukaryotic cell lines
☒	☐ Palaeontology and archaeology
☐	☒ Animals and other organisms
☒	☐ Clinical data
☒	☐ Dual use research of concern
☒	☐ Plants

Methods

n/a	Involved in the study
☒	☐ ChIP-seq
☐	☒ Flow cytometry
☒	☐ MRI-based neuroimaging

Antibodies

Antibodies used

CD45 (Cat# 103101; 30-F11; BioLegend), CD3 (Cat# 100311;145-2C11; BioLegend), CD4 (Cat# 100505; RM4-5; BioLegend), CD8 (Cat# 100711; 53-6.7; BioLegend), CD107a (Cat# 121611; 1D4B; BioLegend)
GzmB (Cat# 515405; GB11; BioLegend) and IRF7 (Cat# 12-5829-82; MNGPKL; Invitrogen)

Human
CD4 (Cat# 300501; RPA-T4; BioLegend), CD8 (Cat# 301002; RPA-T8; BioLegend), CD45RA (Cat# 983004; HI100; BioLegend), CD45RO (Cat# 304251; UCHL1; BioLegend), CD44 (Cat# 103001; IM7; BioLegend), CCR7 (Cat# 988902; G043H7; BioLegend), PD1 (Cat# 329933; EH12.2H7; BioLegend), CD62L (Cat# 980702; DREG-56; BioLegend), IRF7 (Cat# 656003; 12G9A36; BioLegend)

IHC
CD3 (Cat# A0452; Dako), Gr1 (Cat# HM1039; NIMP-R14; Hycult Biotech, Uden), and Mac2 (Car# CL8942AF7; M3/38, Cedarlane, ON, Canada) to quantify leukocytes.
IF staining
CD3 (Cat# A0452; Dako), CD4 (Cat# 100506; RM4-5, BioLegend), and CD8 (Cat# 100775; 53-67, BioLegend)

For flow cytometry, Abs were diluted 1/100, and for IF staining, Abs were diluted 1/200.

Validation

Antibodies used in this study are commercially available and have been validated by the manufacturers. Validation statements are provided on the manufacture's website.
human antibody CD45 HI30 BioLegend
https://www.biolegend.com/de-at/products/purified-anti-human-cd45-antibody-710
human antibody CD3 OKT3 BioLegend
https://www.biolegend.com/de-de/products/purified-anti-human-cd3-antibody-3642
human antibody CD4 RPA-T4 BioLegend
https://www.biolegend.com/de-at/products/purified-anti-human-cd4-antibody-830
human antibody CD8 RPA-T8 BioLegend
https://www.biolegend.com/de-de/products/purified-anti-human-cd8a-antibody-839
human antibody CD69 FN50 BioLegend
https://www.biolegend.com/en-ie/products/pe-anti-human-cd69-antibody-1672
human antibody CD45RA HI100 BioLegend
https://www.biolegend.com/nl-be/products/purified-anti-human-cd45ra-antibody-689
human antibody CD44 IM7 BioLegend
https://www.biolegend.com/en-gb/products/purified-anti-mouse-human-cd44-antibody-318
human antibody GZMB GB11 BioLegend
https://www.biolegend.com/en-ie/products/alexa-fluor-647-anti-human-mouse-granzyme-b-antibody-6067
human antibody IRF7 12G9A36 BioLegend
https://www.biolegend.com/fr-fr/products/pe-anti-irf7-antibody-9243
human antibody CCR7/CD197 Go43H7 BioLegend
https://www.biolegend.com/de-at/products/pe-anti-human-cd197-ccr7-antibody-7498
human antibody CD62L DREG-56 BioLegend
https://www.biolegend.com/fr-ch/products/fitc-anti-human-cd62l-23101

mouse antibody CD45 30-F11 BioLegend
https://www.biolegend.com/nl-be/products/purified-anti-mouse-cd45-antibody-102
mouse antibody CD3 145-2C11 BioLegend
https://www.biolegend.com/fr-lu/products/apc-anti-mouse-cd3epsilon-antibody-21
mouse antibody CD4 RM4-5 BioLegend
https://www.biolegend.com/nl-nl/products/purified-anti-mouse-cd4-antibody-484
mouse antibody CD8 53-6.7 BioLegend
https://www.biolegend.com/nl-be/products/apc-anti-mouse-cd8a-antibody-150
human antibody IRF7 MNGPKL Invitrogen
https://www.thermofisher.com/antibody/product/IRF7-Antibody-clone-MNGPKL-Monoclonal/12-5829-82
mouse antibody CD44 IM7 BioLegend
https://www.biolegend.com/en-gb/products/purified-anti-mouse-human-cd44-antibody-318

Eukaryotic cell lines

Policy information about cell lines and Sex and Gender in Research

Cell line source(s)	HEK293T (https://www.atcc.org/products/crl-3216)
Authentication	not authenticated
Mycoplasma contamination	Cells which tested negative for mycoplasma were used for experiments
Commonly misidentified lines (See ICLAC register)	No commonly misidentified cell lines were used.

Animals and other research organisms

Policy information about studies involving animals; ARRIVE guidelines recommended for reporting animal research, and Sex and Gender in Research

Laboratory animals	Male mice on a C57BL/6 background, aged between 8 and 16 weeks, were used. Mice were maintained in specific pathogen-free conditions with controlled humidity and temperature with a light/dark cycle of 12h each.
Wild animals	not used
Reporting on sex	only males were used
Field-collected samples	No field-collected samples were used in this study.
Ethics oversight	All animal experiments were performed with the approval of the regulatory committee (Free and Hanseatic City of Hamburg, Authority for Justice and Consumer Protection, Office for Consumer Protection, Department of Food Safety and Veterinary Affairs) and carried out in accordance with German animal protection law.

Note that full information on the approval of the study protocol must also be provided in the manuscript.

Plants

Seed stocks	n.a.
Novel plant genotypes	n.a.
Authentication	n.a.

Flow Cytometry

Plots

Confirm that:

- [x] The axis labels state the marker and fluorochrome used (e.g. CD4-FITC).
- [x] The axis scales are clearly visible. Include numbers along axes only for bottom left plot of group (a 'group' is an analysis of identical markers).
- [x] All plots are contour plots with outliers or pseudocolor plots.
- [x] A numerical value for number of cells or percentage (with statistics) is provided.

Methodology

Sample preparation

Single-cell preparations were obtained from kidney and blood samples for human leukocyte analysis. The process involved enzymatic digestion of the kidney tissues with collagenase D at a concentration of 0.4 mg/ml (Roche, Mannheim, Germany) and DNase I at 10 µg/ml (Sigma-Aldrich, Saint Louis, MO) in RPMI 1640 medium at 37°C for 30 minutes, followed by mechanical dissociation using the gentleMACS system (Miltenyi Biotec). Blood samples were processed for separation using Leucosep tubes (Greiner Bio-One, Kremsmünster, Austria). Subsequently, all samples were passed through a 30-µm mesh filter (Partec, Görlitz, Germany) before antibody labeling and flow cytometric analysis.

Murine spleen cells were harvested by pressing the organ through a 70-µm cell strainer. Red blood cells were removed using a lysis buffer containing 155 mM NH_4Cl, 10 mM $KHCO_3$, and 10 µM EDTA at a pH of 7.2. For isolating lymphocytes from mouse kidneys, the organs were enzymatically digested with 400 µg/ml collagenase D (Roche) and 10 U/ml DNase I (Sigma-Aldrich) at 37°C for 30 minutes, followed by mechanical dissociation using the gentleMACS system (Miltenyi Biotec). Then, leukocytes were isolated through density gradient centrifugation using 37% Easycoll (Merck Millipore) and a subsequent filtration step through a 30-µm cell strainer (Partec).

Instrument

FACS symphony

Software

FlowJo

Cell population abundance

Cell populations were abundant enough for any of the analysis. Over 5000 cells of each target cell population were detected from the kidney, spleen and blood of human and mice.

Gating strategy

Gating strategy is indicated in the text, figures, or figure legends.

- [ ] Tick this box to confirm that a figure exemplifying the gating strategy is provided in the Supplementary Information.
